# Evaluation of School-Based Health Education Intervention on the Incidence of Soil-Transmitted Helminths in Pupils of Rural Communities of Eastern Kogi State, North Central Nigeria

**DOI:** 10.1155/2022/3117646

**Published:** 2022-02-26

**Authors:** Clement Ameh Yaro, Ezekiel Kogi, Sodangi Abdulkarim Luka, Luay Alkazmi, Junaidu Kabir, Kenneth Nnamdi Opara, Gaber El-Saber Batiha, Kamba Bayo, Friday Maduka Chikezie, Albert Bamigbade Alabi, Salamat Ibrahim Yunusa

**Affiliations:** ^1^Department of Animal and Environmental Biology, University of Uyo, Uyo, Akwa Ibom State, Nigeria; ^2^Department of Zoology, Ahmadu Bello University, Zaria, Kaduna State, Nigeria; ^3^Biology Department, Faculty of Applied Sciences, Umm Al-Qura University, Makkah 21955, Saudi Arabia; ^4^Department of Veterinary Public Health and Preventive Medicine, Ahmadu University, Zaria, Kaduna State, Nigeria; ^5^Department of Pharmacology and Therapeutics, Faculty of Veterinary Medicine, Damanhour University, Damanhour 22511, AlBeheira, Egypt; ^6^Neglected Tropical Disease Control Program, Kogi State Ministry of Health, Lokoja, Kogi State, Nigeria

## Abstract

The negative impact of soil-transmitted helminths (STHs) in Nigeria is enormous, and it poses serious public health issues and concerns. This study was undertaken to investigate the impact of health education intervention on reinfection of STHs in pupils of rural schools of Kogi East, North Central Nigeria. A total of 10 schools with the highest prevalence of STHs at baseline were selected from the 45 schools assessed during the baseline survey. These 10 schools were randomly paired into two groups of 5 schools per group. Five schools were dewormed and given health education (DHE) intervention while the other 5 schools were dewormed only (DO) without health education. Reassessment of schools for reinfection was carried out for a period of 12 months. Data obtained were analyzed using descriptive statistics. Student's *t*-test was used to make comparison between interventions in the incidence of infections. Analysis was carried out at *p* < 0.05. Reinfection with STHs was observed from the 28^th^ week (7^th^ month) of both interventions with incidence of 0.29 (2 pupils) and 1.00 (7 pupils) in DO and DHE schools, respectively. In the 36^th^ week (9^th^ month), incidence observed in schools given DHE was 0.56 (5 pupils) while incidence of 0.89 (8 pupils) was observed in DO schools, and there was no significant difference (*t* = −1.000, *p* = 0.347) between the interventions. At 48^th^ week (12^th^ month), there was no significant difference (*t* = −0.547, *p* = 0.599) in incidence between the DHE and DO schools with incidence of 1.00 (12 pupils) and 0.83 (10 pupils), respectively. Hookworms had an incidence of 0.78 (7 pupils) at DHE schools and 0.56 (5 pupils) at DO schools in the 36^th^ week while an incidence of 0.92 (11 pupils) and 0.83 (10 pupils) at DHE and DO schools, respectively, in the 48^th^ week. *Ascaris lumbricoides* was only observed in DHE schools in a pupil with an incidence of 0.11 (1 pupil) and 0.08 (1 pupil) at 36^th^ and 48^th^ weeks. There was no significant difference in the prevalence of the parasites between DO and DHE intervention groups (*p* > 0.05). School-based health education intervention had no significant impact on STH incidence in pupils of rural schools in Kogi East. Community-based deworming should be encouraged alongside improvement in the water, sanitation, and hygiene infrastructures and practices at both school and home.

## 1. Introduction

Soil-transmitted helminths (STHs) are among the foremost causes of global health problems especially in underprivileged and deprived populations where implementation and control are challenging to sustain. Soil-transmitted helminthiasis are caused by parasitic nematodes transmitted through contact with parasites eggs (*Ascaris lumbricoides* and *Trichuris trichiura*) or larvae (hookworms) and are responsible for more than 40% of worldwide morbidity from all tropical infections [1–3]. An estimated 2 billion people are infected worldwide with 819, 439, and 439 million people infected with *A. lumbricoides*, *T. trichiura,* and hookworms, respectively [3, 4].

Soil-transmitted helminthiasis is the most widespread neglected tropical diseases (NTDs) in Nigeria [2]. Children in rural areas lacking clean water and sanitation infrastructures are the most affected [5]. Infection with these parasites leads to hampered cognitive and physical development and nutritional effects [6–8]. Infections with *A. lumbricoides* can cause abdominal pain, lactose intolerance, and decreased absorption of vitamin A and other nutrients. Severe infection with whipworm leads to inflammation at the site of attachment in the intestines and results in colitis and rectal prolapse. Infections with hookworms may lead to intestinal blood loss that results in irondeficiency anaemia [7, 9–12].

Preventive chemotherapy is recommended by WHO as means of controlling STH infections which involves consistent administration of drugs to population at risk. The WHO recommends annual deworming of preschool-aged children and school-aged children in areas where the prevalence of STH is between 20% and 50% and biannual if above 50% are infected [13]. By treating the highest risk group, environmental contaminations are reduced and consequently, infections in the community will decrease [14]. Despite this repetitive treatment, infection prevalence and intensity have rapidly bounced back.

In Nigeria, the main strategy for control of soil-transmitted helminth (STH) infections is the periodic mass drug administration (MDA) of antihelminthics to the population at risk [2]. MDA alone as an intervention does not prevent reinfection of STHs [15, 16]. Therefore, there is need for complementary measures to prevent reinfection. Such measures include health education, good hygiene practices, and improved environmental sanitation. This will help augment the control approach and enhance the effectiveness of MDA for optimal productivity and sustainability [16]. This integrative approach will help reduce the number of treatment rounds, lessen the disease burden, and create a long-standing sustainable control measure.

Health education is a vital, low-cost, and simple component of most interventions for prevention and control of many NTDs. Since NTD transmission is enabled by human activities and behaviour, education on sanitation, and and personal and good hygiene practices can prevent reinfection within the school and community. Health education has been found to reduce the cost of deworming and increase the level of overall health benefits and acceptability of deworming intervention within the community [17–20]. This study was therefore carried out to investigate the impact of school-based health education on the incidence of soil-transmitted helminths in Kogi East, North Central Nigeria.

## 2. Methods

### 2.1. Study Area

**Figure 1:**
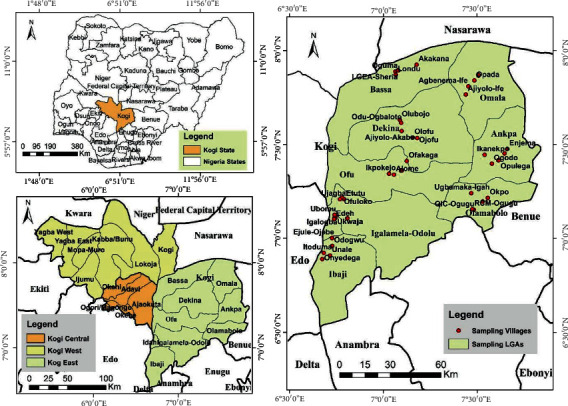
Sampled villages in Kogi East Senatorial District, Nigeria (source: Map Gallery, Geography Department, Ahmadu Bello University, Zaria).

Kogi East is located in Kogi State, North Central Nigeria. It is a geographical region comprising of nine (9) local government areas (LGAs); Ankpa, Bassa, Dekina, Ibaji, Idah, Igalamela/Odolu, Ofu, Olamaboro, and Omala (Figure 1). The district is located between latitude 6°32′33.8′′N to 8°02′44.8′′N and longitude 6°42′08.5′′E to 7°51′50.3′′E [21]. The district occupies an area of 26,197 square kilometres sharing boundaries with six (6) states of Nigeria. To the North, it shares boundaries with Nassarawa, to the West with Edo and Delta States, while to the East by Benue, Anambra, and Enugu States [22]. The district is located in the Southern Guinea Savanna vegetation belt of Nigeria, characterized by a wet season from April to October and a dry season from November to March with an annual rainfall ranged between 800 and 1100 mm. The average annual temperature ranged between 24.1 and 31.2°C [23].

### 2.2. Ethical Approval

Prior to commencement of the study, ethical clearance was also obtained from Research Ethics Committee, Kogi State Ministry of Health (KSMoH), Lokoja, with reference number MOH/KGS/1376/1/82, and permission to carry out the study in schools was obtained from the State Universal Basic Education Board (SUBEB), Lokoja, with reference number KG/SUBEB/GEN/04/‘T' which was conveyed to the education secretaries and head teachers of the schools. Also, the study was approved by the Committee on Human Subjects for Research with reference number ABUCAUC/2021/003.

### 2.3. Inclusion and Exclusion Criteria

Children attending schools in rural communities of Kogi East with ages from 5 to 15 years were included in this study. Preschool-aged children (less than 5 years) and children older than 15 years attending rural schools in Kogi East were not included in this study.

### 2.4. School Mobilization and Sensitization

Advocacy visits were paid to the Honourable Commissioner for Health, and this was preceded by letters from the KSMoH and also the SUBEB to the Education Secretaries of the Local Government Education Authorities (LGEAs). They were adequately briefed about the purpose of the study. Thereafter, schools selected for the study were visited and mobilized for the study.

### 2.5. Study Design/Selection of Endemic Schools for Intervention Studies

Prior to the intervention study, a baseline survey on the prevalence of STHs was carried out in 45 schools in rural communities of the 9 LGAs (Ankpa, Bassa, Dekina, Ibaji, Idah, Igalamela/Odolu, Ofu, Olamaboro, and Omala) of Kogi East Senatorial District from January 2018 to June 2018 (Table 1) [21].

**Figure 2:**
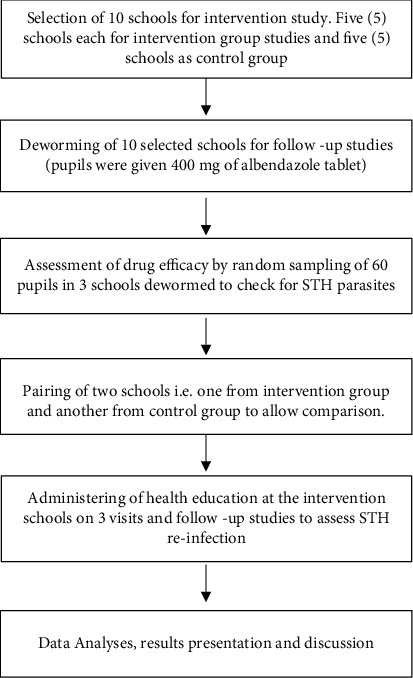
Flow chart of study procedure.

The intervention study commenced from January 2018 to December 2019. To evaluate the effect of health education on the incidence of STHs, a total of 10 schools with the highest prevalence from the baseline study were selected. These 10 schools were divided into two (2) groups of five schools per group. The first group of five (5) schools served as the intervention group, while the second group of five (5) schools served as the control group. Schools from the two groups were randomly paired (Figure 2).

### 2.6. Sample Size and Selection of Participating Children

Sample size was determined for the baseline study (prevalence studies) but for the intervention studies, the baseline prevalence was used to determine the sample size. Only school children of age 5–15 years who consented to partake in the 12-month follow-up study were included.

### 2.7. Statement of Consent from Participants

The guardians/parents of the children were informed about the purpose, objectives, and benefits of the study. Written consents were obtained from the guardians/parents of study participants, informing them of their rights and granting permission for their children to participate in the study. A total of 324 pupils consented to participate in the follow-up study.

### 2.8. Deworming of School Children for Follow-Up Studies

All school children in the 10 selected schools were given a 400 mg chewable albendazole tablet (manufactured and donated by GlaxoSmithKline to World Health Organization) by trained health officials from the Kogi State Ministry of Health. The essence of deworming all school children was to avoid transmission from nonparticipating children to participating children. The tablets used for deworming of the school children were provided by the NTDs Unit, Kogi State Ministry of Health, Lokoja, Kogi State, Nigeria. During the deworming, each child was monitored to ensure that the tablet is chewed and swallowed. Efficacy of the albendazole treatment was assessed in a random sampling of 60 pupils each from 3 schools dewormed to check for the presence of at least one of the STH species [24].

### 2.9. Health Education Intervention

The health education protocol was administered during every visit at each intervention school, and it consists of two components. First, pupils were taught on STH infections, transmission, and prevention. Urban School Health Kit by WHO [25] was adopted during this component. During this intervention, pupils were taught on ways to improve their personal hygiene and understand the importance of preventing STH infection. Secondly, workshop was organized for teachers and staff of each school with the goal of promoting an integrated health program. These workshops were held following deworming.

Posters highlighting key health messages were displayed at strategic locations around the school and in classrooms while brochures were distributed to teachers and staff. The key messages for prevention used in this study were washing hands before eating, washing hands with soap after playing with soil, washing hands with soap after using the toilet, wearing slippers or shoes when going outside, avoiding open (indiscriminate) defecation, washing vegetables and fruits before consumption, drinking clean (boiled) water, covering food from flies, and cutting nails periodically.

Proper attention was given to the school children in the health education intervention group to ensure they clearly understood the health education messages by asking them questions to assess their knowledge on the subject matter. This was repeated on every visit to the intervention schools.

### 2.10. Follow-Up Assessments

**Figure 3:**
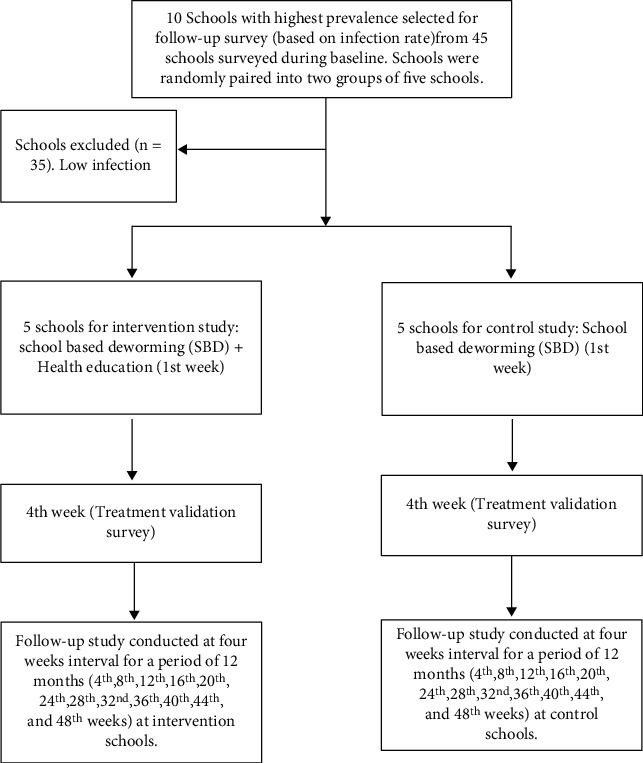
Flow chart of intervention procedure. Note: during each follow-up study, stool specimens were collected and examined. At the intervention schools, health education rehearsal was carried out.

The follow-up assessments commenced one month after deworming and were carried out monthly throughout the 12 months of the study (Figure 3). The assessment was conducted at 4-week interval to enable us monitor the month in which reinfection occurred.

### 2.11. Sample Collection and Parasitological Examination

Stool samples were collected at 4-week interval from the 324 school children who voluntarily agreed to take part throughout the 12 months of the study. Each child in the study was given a clean specimen bottle to take home. The pupils were adequately instructed on how to collect the stool specimen. A single faecal sample was collected from each child and preserved using 10% formalin. Stool samples collected were taken to the Laboratory of the Department of Animal and Environmental Biology, Kogi State University, Anyigba, and processed within 48 hours of collection. Parasitological examination was carried out using formal ether sedimentation technique as described by Gracia [26] and Abossie and Seid [27]. Briefly described, 1 g emulsified faeces was mixed with 7 ml of 10% formal water and then mixed and sieved in another tube. Then, 3 ml of ether was added and centrifuged immediately at 3000 rpm for 1 min. Finally, the supernatant was discarded, and then, small portion of the sediment was transferred to a slide, stained with iodine, covered with cover slip, and examined microscopically for parasites eggs and larvae. Two slides were prepared per sample.

### 2.12. Statistical Analyses

Data were entered using Microsoft Excel version 2013.Descriptive statistics were used to compute prevalence and incidence.The Student *t*-test was used to determine the level of significance between the intervention group and the control group.All analyses were performed using the Statistical Package for Social Sciences (SPSS) software (Version 22.0 for Windows; SPSS Inc., Chicago, IL, USA).

(1) $$\begin{array}{c} \text{Incidence}=\frac{\text{number of new cases }}{\text{time }\left(\text{months}\right)}, \end{array}$$

(2) $$\begin{array}{c} \text{Point prevalence }\left(\%\right)=\frac{\text{number of current cases }}{\text{population at the same specified point in time}}\times 100 \end{array}$$

## 3. Results

A total of 2,331 pupils were dewormed in 10 schools across Kogi East (Table 2). School children from five of the dewormed schools were subjected to health education. The effect of both interventions was assessed over a 12-month period.

**Figure 4:**
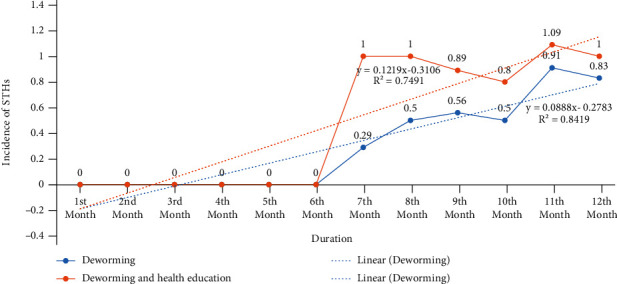
Monthly incidence of STHs in DO and DHE schools in Kogi East, Nigeria.

No parasitic infection was observed up to the 24^th^ week (6^th^ month) of stool examination. Soil-transmitted helminth infections were observed from the 28^th^ week (7^th^ month) after the administration of both interventions, i.e., deworming only (DO) and deworming and health education (DHE). The incidence of STHs at the onset of infection at the 28^th^ week was 0.29 (2 pupils) and 1.00 (7 pupils) for DO and DHE schools, respectively (Table 3 and Figure 4). There was no statistically significant difference (*t* = −2.160, *p* value = 0.063) in incidence of STH infections between interventions at the 28^th^ week.

In the 36^th^ week (9^th^ month), incidence of 0.89 (8 pupils) was observed in school children given DHE which was higher than incidence of 0.56 (5 pupils) in school children in DO schools, and there was no statistically significant difference (*t* = −1.000, *p* value = 0.347) between both interventions (Tables 3 and 4 and Figure 4). Also, at the 48^th^ week (12^th^ month), the incidence of 1.00 (12 pupils) and 0.83 (10 pupils) was observed in DHE and DO schools, respectively, and there was no statistically significant difference (*t* = −0.547, *p* value = 0.599) in the incidence between the interventions (Tables 3 and 4 and Figure 4).

The incidence of individual parasites was statistically not significant at the 36^th^ and 48^th^ weeks between interventions. At the 36^th^ week, the incidence of *A. lumbricoides* in school children was 0.11 (1 pupil) at DHE schools while the incidence of hookworms was 0.78 (7 pupils) and 0.56 (5 pupils) at DHE and DO schools, respectively (Tables 5 and 6).

At the 48^th^ week, the incidence of *A. lumbricoides* in school children was 0.08 (1 pupil) at DHE schools while the incidence of hookworms was 0.92 (11 pupils) and 0.83 (10 pupils) at DHE and DO schools, respectively (Tables 5 and 6).

Comparison of incidence of infection between DHE and DO schools at 12^th^, 24^th^, 36^th^, and 48^th^ weeks revealed no significant difference (*p* > 0.05), although the incidence was higher in the DHE than DO schools.

During the follow-up, *T. trichiura* and *S. stercoralis* were not observed in school children throughout.

## 4. Discussion

The present study assessed the impact of health education on the incidence of STHs among pupils of rural primary schools in Kogi East, Kogi State, Nigeria. The study revealed that health education has no significant effect on the reinfection of soil-transmitted helminths in the region. Chemotherapy proves to be more effective than health education.

The observation of this study contrasts the series of studies previously conducted on the effect of health education on the prevalence of STHs elsewhere [24, 28, 29], where it was reported that health education caused a reduction in parasitic infection but is similar to the findings of a study in Ethiopia [30] where a prevalence of 25.8% at baseline and an incidence of 23.8% at endline were reported. The prevalence of STH infections was not significantly decreased at the endline compared with the baseline (PR = 0.92, 95%CI = (0.62, 1.38)). Gizaw et al. [30] reported that water, sanitation, and hygiene (WASH) education was significantly associated with households' sanitation performance. They stated that health education increases the awareness on good WASH practices and encourages behavioural changes especially when carried out at the household level rather than at school level for better performance. The health education intervention in India [28] and Mali [29] was effective because it was a community-based total sanitation approach while in this study, school-based approach was used; hence, this might have accounted for the variance in result obtained. A study in Malaysia [31] and another study in a low- and middle-income country [32] stated that community-based health education intervention is one of the most effective WASH promotion approaches to empower rural communities. A study in Peruvian Amazon [24] recommended that school-based periodic deworming programs are likely to perform better when enhanced with a sustained health hygiene education in an integrated manner.

Health education increases awareness about the potential health implications. The implementation barriers at household level are important factors that need proper consideration as this will subsequently affect the reinfection of these parasites. Several household barrier factors such as financial status, parent education level, culture, and willingness to adhere to instructions should be put under consideration [33–35].

Hookworm recorded highest incidence compared to *A. lumbricoides* and *S. stercoralis*. Previous studies have found that health education has only a minimal, insignificant effect on hookworm infections [24, 36]. Documented reports have shown that children in underprivileged communities are faced with several barriers which affects the positive changes provided by the health education; such factors include lack of financial resources to purchase a pair of shoes and ignorance of parents [37, 38]. In this study, oral interview with the pupils revealed that some of them had one pair of rubber sandals which was used only when going to school and were prevented by their parents from using such rubber sandals at home or when moving around in the village. This practice predisposes them to infections with hookworm and other environmental hazards. Similar observation has been reported by Al-Delamy et al. [31].

The significant reduction in incidence during follow-up observed in this study might be due to chemotherapy administered prior to follow-up study. Series of studies have reported the effectiveness of chemotherapy in control of STH infection especially when done annually. A study in Gurage Zone, Ethiopia [39], reported that chemotherapy results in substantial reduction in overall prevalence and intensity of STHs. The residual infections with STHs in this study are a reflection of the maintenance of transmission among the untreated populations in the community which are constantly in contact with the dewormed children. Some studies in Kenya reported low prevalence of STHs among all age groups given school-based deworming medicines [40–42].

One remarkable finding in this study is the apparent lack of impact of health education on the prevalence of STHs [43]. In contrast, other studies elsewhere [24, 28, 29] have reported an appreciable decrease in parasitic infection. Although the study participants were willing and adherent to some of the health education protocols at intervention, it was observed that poverty and ignorance were major factors hindering the sustainability of health education protocols, and most of the pupils complained that their parents do not have enough money to procure some of the items required for health education strategies. In addition, lack of toilet facilities and potable water in the schools encourage open air defecation which is verifiable source of reinfection.

This study was purely school-based and was able to reveal that short-term health education does not have significant impact on the incidence of STHs. Meanwhile, this study is limited due to its inability to capture children not enrolled in schools thereby allowing spill over effect between enrolled and unenrolled children.

Control of STH relies on preventive chemotherapy to reduce occurrence, severity, and long-term consequences of morbidity, while health education is to reduce risk behaviour and improvement on environmental conditions. The 54^th^ World Health Assembly published the resolution WHA54.19 on STH and SCH [44] and urged members to implement and sustain control activities but with the lack of health education impact on the incidence of STH in this present study, WHO resolution becomes a mirage. It is suggested that government of member states should ensure the adequate provision of good sanitation facilities and basic hygiene infrastructures in schools, if the control and subsequent elimination of STH is to be achieved.

## 5. Conclusions

Health education had no significant effect on the reinfection of soil-transmitted helminths in the Kogi East, North Central Nigeria. Inclusion of health education alongside with both school-based deworming proved not effective than school-based deworming alone. The use of community-based deworming alongside improvement in the water, sanitation, and hygiene infrastructures both at schools and home will be of tremendous benefit in stemming the tide of infection and reinfection of STHs.

## 6. Limitation of Study

The study was purely school-based, and therefore, children and preschool children within the communities that are not registered in schools were not included in the study. We therefore recommend further studies should be conducted on all children within the communities including those not registered in schools.

## Figures and Tables

**Table 1:** Prevalence of STHs in rural primary schools of Kogi East, Nigeria, during baseline survey.

LGAs	Schools (*n*)	Latitude	Longitude	Number positive (prevalence in %)			
				STHs	*A. lumbricoides*	Hookworms	*S. stercoralis*
Ankpa	Ikanekpo (21)	7.4440	7.5398	8 (38.1)	8 (38.1)	0 (0)	0 (0)
	Opulega (25)	7.4146	7.6137	5 (20.0)	0 (0)	5 (20.0)	0 (0)
	Ogodo (37)	7.3964	7.5786	6 (16.2)	1 (2.7)	4 (10.8)	1 (2.7)
	Enokpoli (11)	7.4508	7.6454	1 (9.1)	0 (0)	1 (9.1)	0 (0)
	Enjema (18)	7.4746	7.6599	3 (16.7)	3 (16.7)	0 (0)	0 (0)
Bassa	Akakana (29)	7.9227	7.1777	3 (10.3)	2 (6.9)	1 (3.4)	0 (0)
	Oguma (31)	7.8864	7.0644	6 (19.4)	1 (3.2)	5 (16.1)	0 (0)
	Sheria 1 (36)	7.8920	7.0764	3 (8.3)	1 (2.8)	2 (5.6)	0 (0)
	Sheria 2 (26)	7.8914	7.0798	4 (15.4)	4 (15.4)	0 (0)	0 (0)
	Londu (28)	7.8687	7.0664	6 (21.4)	5 (17.9)	4 (14.3)	0 (0)
Dekina	Olubojo (27)	7.6298	7.0880	7 (25.9)	0 (0)	7 (25.9)	2 (7.4)
	Ojofu (20)	7.5328	7.1806	0 (0)	0 (0)	0 (0)	0 (0)
	Ajiyolo-Akabe (30)	7.5702	7.0976	0 (0)	0 (0)	0 (0)	0 (0)
	Odu-Ogbaloto (35)	7.6138	7.0950	5 (14.3)	2 (5.7)	5 (14.3)	2 (5.7)
	Olofu (31)	7.5358	7.1561	6 (19.4)	0 (0)	6 (19.4)	0 (0)
Ibaji	Itoduma (36)	6.9232	6.6836	2 (5.6)	0 (0)	1 (2.8)	1 (2.8)
	Onyedega (40)	6.8902	6.6755	5 (12.5)	2 (5.0)	3 (7.5)	0 (0)
	Unale (40)	6.9093	6.7167	3 (7.5)	1 (2.5)	1 (2.5)	1 (2.5)
	Ejule-Ojebe (40)	7.0019	6.7278	2 (5.0)	2 (5.0)	0 (0)	0 (0)
	Odogwu (41)	6.9603	6.7288	2 (4.9)	0 (0)	2 (4.9)	0 (0)
Idah	Ukwaja (26)	7.1089	6.7454	11 (42.3)	0 (0)	11 (42.3)	1 (3.8)
	Igalogba (24)	7.1206	6.7476	3 (12.5)	0 (0)	3 (12.5)	0 (0)
	Sabon Gari (21)	7.1091	6.7403	4 (19.0)	0 (0)	4 (19.0)	0 (0)
	Ede (29)	7.1014	6.7386	2 (6.9)	0 (0)	3 (10.3)	1 (3.4)
	Ubomu (24)	7.1252	6.7413	2 (8.3)	0 (0)	2 (8.3)	0 (0)
Igalamela	Ogbogbo 1 (29)	7.1059	6.8088	2 (6.9)	0 (0)	1 (3.4)	0 (0)
	Ogbogbo 2 (22)	7.1064	6.8101	6 (27.3)	0 (0)	5 (22.7)	1 (4.5)
	Etutu (36)	7.2082	6.7921	5 (13.9)	1 (2.8)	4 (11.1)	0 (0)
	Ofuloko (20)	7.2163	6.7811	7 (3.2)	1 (5.0)	6 (30.0)	0 (0)
	Ujagba (9)	7.2082	6.7695	2 (22.2)	0 (0)	0 (0)	2 (22.2)
Ofu	Ejule 1 (25)	7.3604	7.0940	4 (16.0)	1 (4.0)	3 (12.0)	1 (4.0)
	Alome (22)	7.3385	7.0570	6 (27.3)	0 (0)	6 (27.3)	0 (0)
	Ejule (40)	7.3609	7.0985	16 (40.0)	14 (35.0)	3 (7.5)	0 (0)
	Ikpokejo-Umomi (20)	7.3435	7.0308	2 (10.0)	0 (0)	2 (10.0)	0 (0)
	Ofakaga (30)	7.4103	7.1250	4 (13.3)	2 (6.7)	2 (6.7)	0 (0)
Olamaboro	Ogugu 1 (35)	7.1553	7.4750	1 (2.9)	1 (2.9)	0 (0)	0 (0)
	Ogugu (36)	7.1518	7.4823	3 (8.3)	1 (2.8)	2 (5.6)	0 (0)
	Okpo (39)	7.2153	7.5570	8 (20.5)	2 (5.1)	4 (10.3)	1 (2.6)
	Ugbamaka-Igah (24)	7.2404	7.4688	5 (20.8)	0 (0)	5 (20.8)	0 (0)
	Igah-Ikeje (20)	7.1959	7.5296	4 (20.0)	0 (0)	4 (20.0)	0 (0)
Omala	Central Abejukolo (40)	7.8688	7.5061	9 (22.5)	0 (0)	9 (22.5)	0 (0)
	Opada (19)	7.8386	7.4853	7 (36.8)	0 (0)	7 (36.8)	0 (0)
	Agbenema-Ife (40)	7.8070	7.4533	20 (50.0)	0 (0)	20 (50.0)	0 (0)
	Islamiya Abejukolo (40)	7.8644	7.5074	12 (30.0)	1 (2.5)	11 (27.5)	0 (0)
	Ajiyolo-Ife (23)	7.7637	7.4399	0 (0)	0 (0)	0 (0)	0 (0)
	Overall (1295)			222 (17.1)	56 (4.3)	164 (12.7)	14 (1.1)

*n*: number examined (source: Yaro et al. [21]: baseline study).

**Table 2:** Deworming of primary school children in Kogi East.

S/No.	School name	LGA	Number of pupils dewormed	Interventions
1	LGEA Agbenema	Omala	235	He+SBD
2	LGEA Opada	Omala	128	SBD
3	LGEA Islamiya Abejukolo	Omala	132	He+SBD
4	LGEA Central Abejukolo	Omala	371	SBD
5	St. Martins de Porres, Ejule	Ofu	326	He+SBD
6	LGEA Alome	Ofu	190	SBD
7	LGEA Ogbogbo	Igalamela	234	He+SBD
8	LGEA/QIC Ukwaja	Idah	184	SBD
9	LGEA Olubojo	Dekina	128	He+SBD
10	All Saint Ikanekpo	Ankpa	403	SBD
Total			2,331	

HE+SBD: health education and school-based deworming; SBD: school-based deworming.

**Table 3:** Comparison of monthly incidence of STHs during 12 months of follow-up in Kogi East, Nigeria.

LGAs/intervention	Schools	n	New cases (incidence of STHs per month during follow-up)											
			4^th^ week (1st month)	8^th^ week (2nd month)	12^th^ week (3rd month)	16^th^ week (4th month)	20^th^ week (5th month)	24^th^ week (6th month)	28^th^ week (7th month)	32^nd^ week (8th month)	36^th^ week (9th month)	40^th^ week (10th month)	44^th^ week (11th month)	48^th^ week (12th month)
Deworming only														
Ankpa	Ikanekpo	42	0 (0.00)	0 (0.00)	0 (0.00)	0 (0.00)	0 (0.00)	0 (0.00)	0 (0.00)	2 (0.25)	2 (0.22)	2 (0.20)	4 (0.36)	4 (0.33)
Idah	Ukwaja	32	0 (0.00)	0 (0.00)	0 (0.00)	0 (0.00)	0 (0.00)	0 (0.00)	0 (0.00)	0 (0.00)	0 (0.00)	0 (0.00)	1 (0.09)	1 (0.08)
Ofu	Alome-Umomi	24	0 (0.00)	0 (0.00)	0 (0.00)	0 (0.00)	0 (0.00)	0 (0.00)	0 (0.00)	0 (0.00)	1 (0.11)	1 (0.10)	2 (0.18)	2 (0.17)
Omala	Opada	24	0 (0.00)	0 (0.00)	0 (0.00)	0 (0.00)	0 (0.00)	0 (0.00)	1 (0.14)	1 (0.13)	1 (0.11)	1 (0.10)	2 (0.18)	2 (0.17)
Omala	Central Abejukolo	35	0 (0.00)	0 (0.00)	0 (0.00)	0 (0.00)	0 (0.00)	0 (0.00)	1 (0.14)	1 (0.13)	1 (0.11)	1 (0.10)	1 (0.09)	1 (0.08)
	Total	157	0 (0.00)	0 (0.00)	0 (0.00)	0 (0.00)	0 (0.00)	0 (0.00)	2 (0.29)	4 (0.50)	5 (0.56)	5 (0.50)	10 (0.91)	10 (0.83)
Deworming and health education														
Dekina	Olubojo	26	0 (0.00)	0 (0.00)	0 (0.00)	0 (0.00)	0 (0.00)	0 (0.00)	0 (0.00)	0 (0.00)	0 (0.00)	0 (0.00)	1 (0.09)	1 (0.08)
Igalamela	Ogbogbo 2	30	0 (0.00)	0 (0.00)	0 (0.00)	0 (0.00)	0 (0.00)	0 (0.00)	2 (0.29)	2 (0.25)	2 (0.22)	2 (0.20)	3 (0.27)	3 (0.25)
Ofu	Ejule 2	40	0 (0.00)	0 (0.00)	0 (0.00)	0 (0.00)	0 (0.00)	0 (0.00)	1 (0.14)	1 (0.13)	1 (0.11)	1 (0.10)	2 (0.18)	2 (0.17)
Omala	Agbenema-Ife	36	0 (0.00)	0 (0.00)	0 (0.00)	0 (0.00)	0 (0.00)	0 (0.00)	2 (0.29)	3 (0.38)	3 (0.33)	3 (0.30)	4 (0.36)	4 (0.33)
Omala	Islamiya-Abejukolo	35	0 (0.00)	0 (0.00)	0 (0.00)	0 (0.00)	0 (0.00)	0 (0.00)	2 (0.29)	2 (0.25)	2 (0.22)	2 (0.20)	2 (0.18)	2 (0.17)
	Total	167	0 (0.00)	0 (0.00)	0 (0.00)	0 (0.00)	0 (0.00)	0 (0.00)	7 (1.00)	8 (1.00)	8 (0.89)	8 (0.80)	12 (1.09)	12 (1.00)
*t*-test was calculated between the two interventions		*t*	NA	NA	NA	NA	NA	NA	-2.160	-1.257	-1.000	-1.000	-0.535	-0.547
		df	NA	NA	NA	NA	NA	NA	8	8	8	8	8	8
		*p* value	NA	NA	NA	NA	NA	NA	0.063 ns	0.244 ns	0.347 ns	0.347 ns	0.608 ns	0.599 ns

*n*: number examined; ns: not significant at *p* > 0.05; NA: not available.

**Table 4:** Incidence of STHs during follow-up at 12^th^, 24^th^, 36^th^, and 48^th^ weeks for both interventions.

LGAs/intervention	Schools	*n*	New cases (point prevalence in %) (incidence of STHs per month during follow-up)			
			12^th^ week (3 months)	24^th^ week (6 months)	36^th^ week (9 months)	48^th^ week (12 months)
Deworming only						
Ankpa	Ikanekpo	42	0 (0.0)(0.00)	0 (0.0)(0.00)	2 (4.8)(0.22)	4 (9.5)(0.33)
Idah	Ukwaja	32	0 (0.0)(0.00)	0 (0.0)(0.00)	0 (0.0)(0.00)	1 (3.1)(0.08)
Ofu	Alome-Umomi	24	0 (0.0)(0.00)	0 (0.0)(0.00)	1 (4.2)(0.11)	2 (8.3)(0.17)
Omala	Opada	24	0 (0.0)(0.00)	0 (0.0)(0.00)	1 (4.2)(0.11)	2 (8.3)(0.17)
Omala	Central Abejukolo	35	0 (0.0)(0.00)	0 (0.0)(0.00)	1 (2.9)(0.11)	1 (2.9)(0.08)
	Total	157	0 (0.0)(0.00)	0 (0.0)(0.00)	5 (3.2)(0.56)	10 (6.4)(0.83)
Deworming and health education						
Dekina	Olubojo	26	0 (0.0)(0.00)	0 (0.0)(0.00)	0 (0.0)(0.00)	1 (3.9)(0.08)
Igalamela	Ogbogbo 2	30	0 (0.0)(0.00)	0 (0.0)(0.00)	2 (6.7)(0.22)	3 (10.0)(0.25)
Ofu	Ejule 2	40	0 (0.0)(0.00)	0 (0.0)(0.00)	1 (2.5)(0.11)	2 (5.0)(0.17)
Omala	Agbenema-Ife	36	0 (0.0)(0.00)	0 (0.0)(0.00)	3 (8.3)(0.33)	4 (11.1)(0.33)
Omala	Islamiya Abejukolo	35	0 (0.0)(0.00)	0 (0.0)(0.00)	2 (5.7)(0.22)	2 (5.7)(0.17)
	Total	167	0 (0.0)(0.00)	0 (0.0)(0.00)	8 (4.8)(0.89)	12 (7.2)(1.00)
	*t*-test was calculated between the two interventions	*t*	NA	NA	-1.000	-0.547
		df	NA	NA	8	8
		*p* value	NA	NA	0.347 ns	0.599 ns

*n*: number examined; STHs: soil-transmitted helminths; ns: not significant at *p* > 0.05; NA: not available.

**Table 5:** Comparison of incidence of parasites species at 12^th^, 24^th^, 36^th^, and 48^th^ weeks in Kogi East, Nigeria.

LGAs	Schools	Incidence at 12th week (3^rd^ month)				Incidence at 24th week (6^th^ month)				Incidence at 36th week (9^th^ month)				Incidence at 48th week (12^th^ month)			
		*n*	A	H	S	*n*	A	H	S	*n*	A	H	S	*n*	A	H	S
Deworming only (DO)																	
Ankpa	Ikanekpo	42	0 (0.00)	0 (0.00)	0 (0.00)	37	0 (0.00)	0 (0.00)	0 (0.00)	39	0 (0.00)	2 (0.22)	0 (0.00)	42	0 (0.00)	4 (0.33)	0 (0.00)
Idah	Ukwaja	38	0 (0.00)	0 (0.00)	0 (0.00)	34	0 (0.00)	0 (0.00)	0 (0.00)	34	0 (0.00)	0 (0.00)	0 (0.00)	32	0 (0.00)	1 (0.08)	0 (0.00)
Ofu	Alome-Umomi	34	0 (0.00)	0 (0.00)	0 (0.00)	34	0 (0.00)	0 (0.00)	0 (0.00)	28	0 (0.00)	1 (0.11)	0 (0.00)	24	0 (0.00)	2 (0.17)	0 (0.00)
Omala	Opada	38	0 (0.00)	0 (0.00)	0 (0.00)	36	0 (0.00)	0 (0.00)	0 (0.00)	36	0 (0.00)	1 (0.11)	0 (0.00)	24	0 (0.00)	2 (0.17)	0 (0.00)
Omala	Central Abejukolo	40	0 (0.00)	0 (0.00)	0 (0.00)	35	0 (0.00)	0 (0.00)	0 (0.00)	35	0 (0.00)	1 (0.11)	0 (0.00)	35	0 (0.00)	1 0.08)	0 (0.00)
		192	0 (0.00)	0 (0.00)	0 (0.00)	176	0 (0.00)	0 (0.00)	0 (0.00)	172	0 (0.00)	5 (0.56)	0 (0.00)	157	0 (0.00)	10 (0.83)	0 (0.00)
Deworming and health education (DHE)																	
Dekina	Olubojo	38	0 (0.00)	0 (0.00)	0 (0.00)	38	0 (0.00)	0 (0.00)	0 (0.00)	32	0 (0.00)	0 (0.00)	0 (0.00)	26	0 (0.00)	1 (0.08)	0 (0.00)
Igalamela	Ogbogbo 2	38	0 (0.00)	0 (0.00)	0 (0.00)	32	0 (0.00)	0 (0.00)	0 (0.00)	32	0 (0.00)	2 (0.22)	0 (0.00)	30	0 (0.00)	3 (0.25)	0 (0.00)
Ofu	Ejule 2	40	0 (0.00)	0 (0.00)	0 (0.00)	40	0 (0.00)	0 (0.00)	0 (0.00)	40	0 (0.00)	1 (0.11)	0 (0.00)	40	0 (0.00)	2 (0.17)	0 (0.00)
Omala	Agbenema-Ife	40	0 (0.00)	0 (0.00)	0 (0.00)	37	0 (0.00)	0 (0.00)	0 (0.00)	37	1 (0.11)	2 (0.22)	0 (0.00)	36	1 (0.08)	3 (0.25)	0 (0.00)
Omala	Islamiya-Abejukolo	40	0 (0.00)	0 (0.00)	0 (0.00)	38	0 (0.00)	0 (0.00)	0 (0.00)	36	0 (0.00)	2 (0.22)	0 (0.00)	35	0 (0.00)	2 (0.17)	0 (0.00)
		196	0 (0.00)	0 (0.00)	0 (0.00)	185	0 (0.00)	0 (0.00)	0 (0.00)	177	1 (0.11)	7 (0.78)	0 (0.00)	167	1 (0.08)	11 (0.92)	0 (0.00)
	*t*-test		NA	NA	NA		NA	NA	NA		-1.000	-0.784	NA		-1.000	-0.324	NA
	df		NA	NA	NA		NA	NA	NA		8	8	NA		8	8	NA
	*p* value		NA	NA	NA		NA	NA	NA		0.347 ns	0.455 ns	NA		0.347 ns	0.754 ns	NA

*n*: number examined; A: *Ascaris lumbricoides*; H: hookworms; S: *Strongyloides stercoralis*; ns: not significant at *p* > 0.05; NA: not available.

**Table 6:** Comparison of incidence of STHs at 48^th^ week for both interventions according to parasite species.

LGAs	Schools	New cases (point prevalence in %) (incidence per month at 12^th^ month of follow-up)				
		*n*	STHs	A	H	S
Deworming						
Ankpa	Ikanekpo	42	4 (9.5)(0.33)	0 (0.0)(0.00)	4 (9.5)(0.33)	0 (0.0)(0.00)
Idah	Ukwaja	32	1 (3.1)(0.08)	0 (0.0)(0.00)	1 (3.1)(0.08)	0 (0.0)(0.00)
Ofu	Alome-Umomi	24	2 (8.3)(0.17)	0 (0.0)(0.00)	2 (8.3)(0.17)	0 (0.0)(0.00)
Omala	Opada	24	2 (8.3)(0.17)	0 (0.0)(0.00)	2 (8.3)(0.17)	0 (0.0)(0.00)
Omala	Central Abejukolo	35	1 (2.9)(0.08)	0 (0.0)(0.00)	1 (2.9)(0.08)	0 (0.0)(0.00)
		157	10 (6.4)(0.83)	0 (0.0)(0.00)	10 (6.4)(0.83)	0 (0.0)(0.00)
Deworming and health education						
Dekina	Olubojo	26	1 (3.9)(0.08)	0 (0.0)(0.00)	1 (3.9)(0.08)	0 (0.0)(0.00)
Igalamela	Ogbogbo 2	30	3 (10.0)(0.25)	0 (0.0)(0.00)	3 (10.0)(0.25)	0 (0.0)(0.00)
Ofu	Ejule 2	40	2 (5.0)(0.17)	0 (0.0)(0.00)	2 (5.0)(0.17)	0 (0.0)(0.00)
Omala	Agbenema-Ife	36	4 (11.1)(0.33)	1 (2.8)(0.08)	3 (8.3)(0.25)	0 (0.0)(0.00)
Omala	Islamiya Abejukolo	35	2 (5.7)(0.17)	0 (0.0)(0.00)	2 (5.7)(0.17)	0 (0.0)(0.00)
		167	12 (7.2)(1.00)	1 (0.6)(0.08)	11 (6.6)(0.92)	0 (0.0)(0.00)
	*t*-test was calculated between interventions		-0.547	-1.000	-0.324	NA
	df		8	8	8	NA
	*p* value		0.599 ns	0.347 ns	0.754 ns	NA

*n*: number examined; STHs: soil-transmitted helminths; A: *Ascaris lumbricoides*; H: hookworms; S: *Strongyloides stercoralis*; ns: not significant at *p* > 0.05; NA: not available.

## Data Availability

The data sets in this study are available from the corresponding author on reasonable request.

## Abbreviations

STHs: Soil-transmitted helminths
DHE: Dewormed and health education
DO: Dewormed only
STH: Soil-transmitted helminthiasis
MDA: Mass drug administration
NTDs: Neglected tropical diseases
LGEAs: Local government education authorities
SUBEB: State Universal Basic Education Board (SUBEB)
LGA: Local government area
RCF: Relative centrifuge force
CI: Confidence interval.
